# Antibiotic use for respiratory tract infections in nursing homes: a multicentre cross-sectional study

**DOI:** 10.3389/fphar.2025.1721689

**Published:** 2026-01-05

**Authors:** Carl Llor, Rosa Morros, Jesús Mateos-Nozal, Carlota Manuela Zárate Sáez, María N. Vaquero Pinto, Consuelo Rodríguez Jiménez, Carmen Sáez Bejar, Rosa Magallón-Botaya, Priscila Matovelle, Alicia Navarro Sanmartín, Elena López Pérez, Ana García-Sangenís, Ramon Monfà

**Affiliations:** 1 Fundació Institut Universitari per a la Recerca a l’Atenció Primària de Salut Jordi Gol, Barcelona, Spain; 2 CIBER en Enfermedades Infecciosas Instituto Carlos III, Madrid, Spain; 3 Department of Public Health, Research Unit for General Practice, University of Southern Denmark, Odense, Denmark; 4 Universitat Autònoma de Barcelona, Bellaterra, Spain; 5 Geriatrics Department, Hospital Universitario Ramón y Cajal (IRYCIS), Madrid, Spain; 6 Pharmacology Department, Complejo Hospitalario Universitario de Canarias, Santa Cruz de Tenerife, Spain; 7 Internal Medicine Department, Hospital Universitario La Princesa, Instituto de Investigación Sanitaria (IIS-Princesa), Madrid, Spain; 8 Group B21-23R, Health Research Institute of Aragon (IISA), Zaragoza, Spain; 9 Network for Research on Chronicity, Primary Care, and Health Promotion, Zaragoza, Spain; 10 Primary Care Department, Universidad Zaragoza, Zaragoza, Spain; 11 Geriatrics Department, Hospital San Juan de Dios, Zaragoza, Spain; 12 Nursing Home, Aragon Institute of Social Services (IASS), Borja, Spain; 13 Institut Català de la Salut, Barcelona, Spain

**Keywords:** anti-bacterial agents, antimicrobial stewardship, elderly, medical audit, nursing homes, respiratory tract infections

## Abstract

**Introduction:**

To evaluate the use of antibiotic treatment for respiratory tract infections (RTIs) in nursing homes in Spain, assess whether they were indicated based on current guidelines, and identify predictors of both use and inappropriate use of antibiotics.

**Methods:**

Cross-sectional study carried out in 34 nursing homes in five areas in Spain. Nursing staff self-registered residents related to antibiotic use for RTIs in February-April 2023. Nursing staff recorded data on residents with RTIs, including demographics, symptoms, diagnoses, and antibiotic use. Logistic regression models were applied to identify predictors of antibiotic prescription and inappropriate use based on clinical guidelines.

**Results:**

Among 533 recorded RTI cases, the most common diagnoses were common cold (19.7%) and acute bronchitis (18.8%). Antibiotics were prescribed in 77.5% of cases, mainly for otitis media, tonsillitis, and pneumonia, with amoxicillin-clavulanate being the most frequent (34%). Of 328 prescriptions assessed, 63.1% were deemed unnecessary. Factors strongly associated with antibiotic use included confusion (odds ratio 4.72; p = 0.005), perceived demand for antibiotics (OR 3.83; p < 0.001), and fever (OR 3.27; p = 0.005), whereas rhinorrhea reduced the likelihood of prescription (OR 0.23; p < 0.001).

**Discussion:**

The findings reveal substantial overuse of antibiotics for RTIs in nursing homes and highlight the need for targeted interventions to promote guideline-based prescribing and reduce inappropriate use.

## Introduction

In recent years, Europe has experienced a significant increase in the elderly population, reshaping the healthcare landscape as older adults constitute a growing proportion of society. This demographic shift has led to increased demand for long-term care facilities. Globally, the proportion of people over 60 years of age is projected to double between 2015 and 2050, with age-related dependency expected to rise accordingly (Beard et al., 2016). Nursing homes aim to provide a home-like environment, yet they often serve as communal settings where individuals with complex health conditions live in close quarters and share caregivers (Bouza et al., 2023). As a result, nursing home residents are particularly vulnerable to frequent and severe infections, with respiratory and urinary tract infections being the most common (Beckett et al., 2015).

The Antimicrobial Consumption in the European Union (ESAC-Net) report recently updated the prevalence of antimicrobial use in long-term care facilities across 18 European Union countries for the period 2023–2024. The findings indicated an overall prevalence of antimicrobial use of 4.1%, with Spain reporting one of the highest consumption rates at 5.1% (European Centre for Disease Prevention and Control, 2025a). At least 60% of prescriptions issued in nursing homes are estimated to be inappropriate, contributing to the development of multidrug-resistant pathogens, with nearly one-third of these antimicrobials administered prophylactically (Ricchizzi et al., 2018). Additionally, the use of non-first-line antibiotics, especially broad-spectrum ones, exacerbates antimicrobial resistance, leading to higher morbidity, mortality, and societal costs (Vyas et al., 2022). Nursing home residents experience significantly higher rates of antibiotic-resistant infections compared to older adults living independently in the community (Marwick et al., 2013; Xie et al., 2012). This increased risk is parallel by greater antibiotic consumption as individuals of the same age residing in long-term care facilities are prescribed antibiotics more frequently than their community-dwelling counterparts (Kelly et al., 2024). These patterns highlight the unique antimicrobial challenges faced by institutionalized older populations and emphasize the importance of targeted antibiotic stewardship in these settings. The aim of this study is to quantify the prescribing rates of antibiotics for residents with respiratory tract infections (RTIs) in Spanish nursing homes, estimate the proportion of prescriptions that were theoretically not indicated according to current guidelines, and identify the factors that predict both antibiotic use and inappropriate antibiotic use.

## Materials and methods

### Study design

This was a cross-sectional study. For this study, we used the information collected during the first-year registration of a before and after study aimed at evaluating the impact of a multifaceted intervention on antibiotic prescribing among residents in nursing homes (Monfà et al., 2025). The participants who documented the infections were mainly nurses but also doctors, nurse assistants, or nurse helpers in each nursing home.

### Audit registrations

The participants completed the audit form documenting all infections in nursing homes throughout a three-month period, from February to April 2023. This form had two sides. The first side collected general information about the infection, including demographic data, type of infection, details of antibiotic treatment (such as whether antibiotics were administered and, if so, which type), allergies, and referrals to other healthcare settings. The second side of the form gathered detailed information on symptoms, signs, diagnosis, and tests used. In autumn 2022, one nursing home or one ward from a large nursing home in each of the five regions of Spain was recruited for a pilot study to assess whether the template used was understandable. Participants were asked to consecutively register all consultations related to RTIs, but they did not receive any guidance on the definition of as this was designed to be a pragmatic study. However, they were given a simple instruction sheet on how to complete the registration template. Each case was recorded on one line, with participants typically only needing to tick boxes, avoiding written responses.

### Statistical analysis

The potentially unnecessary antibiotic use was defined as this antibiotic given to a patient with a suspected RTI not complying with the minimum criteria for being administered based on national and international guidelines (Huang et al., 2023; European Centre for Disease Prevention and Control, 2025b; Woodhead et al., 2011; Spinks et al., 2021; Stone et al., 2012; Smith et al., 2017; Soler-Cataluña et al., 2022) (Table 1). This analysis was conducted only for patients with a clear diagnosis of RTI. We used chi-square and Student t-tests to compare qualitative and quantitative variables, respectively. We also estimated the patterns of antibiotic prescribing and its appropriateness with logistic regression models. Antibiotic prescription was considered the dependent variable (yes/no). These models controlled for patients’ characteristics, signs and symptoms, as well as for the demand for antibiotics by the resident and/or relatives. Hosmer and Lemeshow goodness of fit and collinearity were calculated. Statistical significance was considered at *P* < 0.05. The data were analyzed with the R v. 4.3 statistical program.

**TABLE 1:** Current recommendations on antimicrobial therapy for residents with respiratory tract infections.

Infection	When to administer antimicrobials	First-line antimicrobial	In case of allergy to penicillin	Alternative antimicrobials	Hospital referral
Common cold	No	Symptomatic	Symptomatic	Symptomatic	Poor progression^a^
Acute pharyngitis or tonsillitis	Only that caused by group A *streptococcus* ^b^	Penicillin V 1.000 mg/8 h, 7–10 d	Clindamycin 300 mg/8 h, 10 d, or any macrolide	Amoxicillin 500 mg/8 h, 10 d, or cefadroxil 500 mg/8 h, 10 d. Amoxicillin and clavulanate 500–125 mg/8 h, 10 d if relapse	Poor clinical evolution^a^, suspicion of locoregional complications or Lemierre’s syndrome. Refer to ENT if cervical adenitis or recurrent peritonsillar abscesses are present
Acute rhinosinusitis	Antibiotics should not be given, except if signs and symptoms do not improve within 10 days, severe symptoms appear from day 3, or symptoms worsen from day 5 onward	Amoxicillin 1 g/8 h, 5 d^c^	Respiratory fluoroquinolone 5 d^d^	Amoxicillin and clavulanate 875–125 mg/8 h, 7–10 d or respiratory fluoroquinolone 7 d if relapse or poor evolution^d^	Persistent fever >39 °C, periorbital oedema, inflammation or erythema, severe headache, neurological focal signs, meningeal signs, confusion, suspected orbital infection, or diplopia. Refer to ENT if chronic infection (>12 weeks) or >4 episodes per year
Acute otitis media	Antibiotics should not be given, except in the presence of risk factors for poor outcomes^e^	Amoxicillin 1 g/8 h, 5 d^c^	Any macrolide	Amoxicillin and clavulanate 875–125 mg/8 h,7–10 d if poor evolution	Chronic otitis (>1 week), mastoiditis, meningitis, facial paralysis, or suspected intracranial complication
Influenza	No	Symptomatic	Symptomatic	Oseltamivir 75 mg/12 h, 5 d^f^	Suspected pneumonia, severe condition, confusion, oxygen saturation <92%, significant comorbidity, respiratory rate ≥30 breaths/min, systolic blood pressure <90 mmHg or diastolic <60 mmhg
Acute bronchitis	In acute bronchitis, antibiotics should not be given, except in specific situations^g^	Symptomatic	Symptomatic	Amoxicillin and clavulanate 500–875/125 mg/8 h, 5 d	Suspected pneumonia, severe condition, confusion, oxygen saturation <92%, significant comorbidity, respiratory rate ≥30 breaths/min, systolic blood pressure <90 mmHg or diastolic <60 mmhg
Pneumonia	Always	Amoxicillin and clavulanate 500–875/125 mg/8 h, 5-7 d^h^ ^,^ ^i^	Respiratory fluoroquinolone 5 d^d^ ^,^ ^h^ ^,^ ^i^	Respiratory fluoroquinolone 5 d^d^ ^,^ ^h^ ^,^ ^i^	Refer to hospital^i^
COPD exacerbation	Only if sputum is purulent and in all cases of very severe COPD exacerbations	Amoxicillin and clavulanate 500 ó 875–125 mg/8 h, 5 d^h^	Respiratory fluoroquinolone 5 d^d^ ^,^ ^h^	Risk of *Pseudomonas* ^j^: ciprofloxacin 750 mg/12 h or levofloxacin 500 mg/12 h, 10 d	Confusion, cyanosis, respiratory rate ≥30 breaths/min, severe dyspnoea, oxygen therapy required, inability to remain at home, or oxygen saturation <92%

^a^ > 2 weeks duration of symptoms, when swallowing is impaired, lingual pharyngitis with airway-digestive tract obstruction, and/or suspected lymphomatous infiltration.

^b^ Antibiotics should be given in immunocompromised patients, those with a history of rheumatic fever, signs of severity (poor general condition, ear pain, and/or severe tonsillar inflammation), and in cases of pharyngitis during a Group A β-hemolytic *streptococcus* outbreak in the facility. In other cases, it is essential to distinguish streptococcal pharyngitis from viral pharyngitis: fever >38.5 °C, absence of cough, pharyngo-tonsillar exudate, and painful cervical adenopathy. If fewer than 2 of these criteria are present, do not give antibiotics; if 2 or more, perform rapid antigen testing (Strep A) and treat based on the result. If Strep A testing is not available, give antibiotics if two or more criteria are met.

^c^ If there is no favorable evolution within 48–72 h, use an alternative antibiotic.

^d^ The use of levofloxacin 500 mg/day is recommended over moxifloxacin 400 mg/day, due to the latter’s potential association with fulminant hepatitis and blistering skin reactions (2008 alert), risk of QT, interval prolongation (2010 alert), and because it should be reserved as second-line treatment for tuberculosis.

^e^ Any of the following: severe symptoms (high fever with intense pain and vomiting), presence of otorrhea, serious underlying disease, recurrent otitis media, bilateral otitis, or tympanic membrane perforation.

^f^ Its use is recommended in any of the following situations: elderly >85 years, frail individuals, and/or those with significant comorbidity.

^g^ Marked deterioration of general condition, frail individuals, and elderly with significant comorbidities.

^h^ Check evolution at 48–72 h. If there is no improvement, refer to hospital.

^i^ Residents under 75 years of age may be treated in the facility and monitored over 48–72 h, provided they do not present any of the following: significant comorbidities, confusion, respiratory rate ≥30 breaths/min, systolic blood pressure <90 mmHg or diastolic <60 mmHg, O_2_ saturation <92%, marked abnormalities on chest X-ray, inability to adhere to treatment in the facility, decompensation of underlying disease, intolerance to oral treatment, or immunosuppression.

^j^ Use of 4 antibiotic regimens in the past year, oral corticosteroids in the last 2 weeks, isolation of *Pseudomonas* in a previous sputum culture, presence of significant bronchiectasis, recent hospitalization, or FEV_1_ <30%.

## Results

### Characteristics of the RTIs

A total of 34 nursing homes participated in the registration period. Nursing staff personnel documented a total of 533 RTIs, of which 346 were in women (64.9%). The median age was 88 years (interquartile range [IQR] 82–92 years). The most frequent diagnosis was common cold, with 105 cases (19.7%), followed by acute bronchitis (100 cases [18.8%]) other RTIs (89 cases [16.7%), and pneumonia (74 residents, 13.9%). Cough was the most frequently observed symptom among residents with RTIs, present in 342 cases (64.2%), followed by increased sputum production and breathlessness, observed in 43.9% and 38.5% of cases, respectively. The most common test performed on these patients was the COVID-19 antigen test, used in 26.6% of cases, while chest X-rays, C-reactive protein rapid tests, and rapid antigen detection tests for streptococcal infection were seldom used (Table 2).

**TABLE 2:** General characteristics of the residents with respiratory tract infections from the nursing homes that completed the registration audit.

Characteristics	Total	Men	Women	*P* value
Number or registrations/infections	533	187	346	​
Age, year, median [IQR]	88 [82, 92]	86 [78, 91]	89 [83, 93]	<0.001
Prior symptom duration, days, median [IQR]	2 [1, 3]	2 [1, 3]	2 [1, 3]	0.894
General signs and symptoms, n (%)				
Fever	136 (25.5)	59 (31.6)	77 (22.3)	0.025
Shaking chills	83 (15.6)	41 (21.9)	42 (12.1)	0.004
Confusion	122 (22.9)	44 (23.5)	78 (22.5)	0.880
Joint and muscle pains	50 (9.4)	20 (10.7)	30 (8.7)	0.542
No general symptoms	261 (49.0)	84 (44.9)	177 (51.2)	0.199
Respiratory tract signs and symptoms, n (%)				
Cough	342 (64.2)	123 (65.8)	219 (63.3)	0.635
Rhinorrhea	138 (25.9)	47 (25.1)	91 (26.3)	0.849
Otorrhea	3 (0.6)	1 (0.5)	2 (0.6)	1.000
Odynophagia	56 (10.5)	15 (8.0)	41 (11.8)	0.220
Tonsillar exudate	18 (3.4)	9 (4.8)	9 (2.6)	0.272
Tender cervical glands	19 (3.6)	5 (2.7)	14 (4.0)	0.568
Dyspnea	205 (38.5)	75 (40.1)	130 (37.6)	0.631
Increase in sputum volume	234 (43.9)	86 (46.0)	148 (42.8)	0.534
Purulent sputum	80 (15.0)	25 (13.4)	55 (15.9)	0.514
Bronchospasm	61 (11.4)	21 (11.2)	40 (11.6)	1.000
None of the above	20 (3.8)	8 (4.3)	12 (3.5)	0.818
Diagnosis, n (%)				
Common cold	105 (19.7)	35 (18.7)	70 (20.2)	0.760
Acute otitis media	3 (0.6)	1 (0.5)	2 (0.6)	1.000
Acute sinusitis	0 (0.0)	0 (0.0)	0 (0.0)	NA
Acute pharyngitis	23 (4.3)	8 (4.3)	15 (4.3)	1.000
Acute tonsillitis	9 (1.7)	5 (2.7)	4 (1.2)	0.344
Acute bronchitis	100 (18.8)	33 (17.6)	67 (19.4)	0.713
Pneumonia	74 (13.9)	21 (11.2)	53 (15.3)	0.241
COPD exacerbation	40 (7.5)	19 (10.2)	21 (6.1)	0.124
Bronchoaspirative RTI	54 (10.1)	25 (13.4)	29 (8.4)	0.095
Influenza	23 (4.3)	5 (2.7)	18 (5.2)	0.251
COVID-19 infection	13 (2.4)	5 (2.7)	8 (2.3)	1.000
Other RTIs^a^	89 (16.7)	30 (16.0)	59 (17.1)	0.860
Tests performed, n (%)				
Rapid antigen detection test	2 (0.4)	0 (0.0)	2 (0.6)	0.765
C-reactive protein rapid test	12 (2.3)	5 (2.7)	7 (2.0)	0.859
Chest X-ray	36 (6.8)	17 (9.1)	19 (5.5)	0.162
COVID-19 test	142 (26.6)	48 (25.7)	94 (27.2)	0.786
No testing	320 (60.0)	110 (58.8)	210 (60.7)	0.743
Antibiotic treatment, n (%)	413 (77.5)	146 (78.1)	267 (77.2)	0.896
Duration of the antibiotic course, days, median [IQR]	7 [7, 7]	7 [7, 7]	7 [7, 7]	0.478
Antibiotic given, n (%)				
Penicillin V	0 (0.0)	0 (0.0)	0 (0.0)	NA
Amoxicillin	15 (2.8)	7 (3.7)	8 (2.3)	0.497
Amoxicillin and clavulanate	181 (34.0)	64 (34.2)	117 (33.8)	1.000
Macrolide or clindamycin	30 (5.6)	12 (6.4)	18 (5.2)	0.701
Cephalosporin	58 (10.9)	17 (9.1)	41 (11.8)	0.406
Fosfomycin	9 (1.7)	1 (0.5)	8 (2.3)	0.243
Trimethoprim + sulfamethoxazole	9 (1.7)	1 (0.5)	8 (2.3)	0.243
Quinolone	91 (17.1)	34 (18.2)	57 (16.5)	0.704
Another antibiotic	25 (4.7)	12 (6.4)	13 (3.8)	0.241

COPD, chronic obstructive pulmonary disease; IQR, interquartile range; NA, not available; RTI, respiratory tract infection.

^a^ Any other RTI, that, according to professional judgment, cannot be classified under any of the above diagnoses.

**FIGURE 1:**
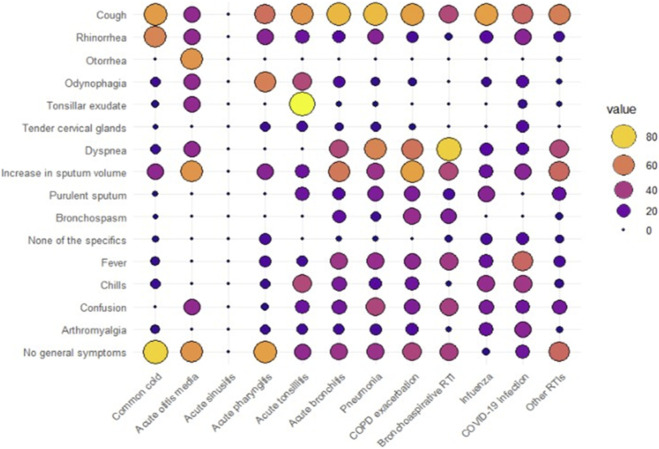
Interaction between symptoms and respiratory tract infection diagnoses.

Figure 1 shows the percentage of symptoms presented by patients for each of the RTI diagnoses. Each column represents a diagnosis and indicates the percentage of symptoms that residents present for each diagnosis. It helps to interpret how each condition is diagnosed. Among the specific respiratory tract symptoms, cough was particularly predominant in the so-called lower RTIs—i.e., acute bronchitis, pneumonia, and chronic obstructive pulmonary disease (COPD) exacerbations—but was also common in cases of flu and common cold. Rhinorrhea was more frequent among residents with common cold, tonsillar exudate in those with acute tonsillitis, and otorrhea in residents with acute otitis media. Dyspnea was highly prevalent in residents with lower RTIs, mainly bronchoaspirative RTIs, and the increase in the volume of expectoration was greater among those with COPD exacerbations and acute bronchitis. Among systemic symptoms, fever was more prevalent in lower RTIs and flu infections, with chills being more prevalent among influenza patients. Confusion was more common in residents with pneumonia and bronchoaspirative RTIs, while arthromyalgias were mostly seen in patients with flu. However, most residents with RTIs presented no general symptoms, except for those with flu infection.

### Antibiotic use for RTIs

As shown in Table 3, antibiotics were administered to 413 residents (77.5%), with slightly higher rates among men compared to women. Certain RTIs were consistently treated with antibiotics, such as acute otitis media and acute tonsillitis. A total of 71 pneumonia episodes (95.9%) were treated with antibacterial agents. Similarly, a high number of cases of acute bronchitis and acute pharyngitis were also associated with high antibiotic prescribing rates, exceeding 90% of cases. Antibiotics were prescribed in 85% of acute COPD exacerbations and 74.1% of bronchoaspirative RTIs. Even in predominantly viral infections—such as COVID-19, influenza, and the common cold—antibiotics were used in 61.5%, 47.8%, and 29.5% of cases, respectively. The most used antibiotic was the combination of amoxicillin and clavulanate, prescribed in 34% of all RTI cases, followed by quinolones and cephalosporins, which accounted for 17.1% and 10.9% of all antibiotics administered, respectively. Notably, amoxicillin alone was used in only 2.8% of infections, and penicillin V was not prescribed in any RTI case (Table 2).

**TABLE 3:** Proportion of residents treated with antibiotics according to diagnosis.

Diagnosis	Total	Men	Women	*P* value
Common cold	31/105 (29.5)	10/35 (28.6)	21/70 (30.0)	0.858
Acute otitis media	3/3 (100.0)	1/1 (100.0)	2/2 (100.0)	1.000
Acute sinusitis	0/0 (0.0)	0/0 (0.0)	0/0 (0.0)	NA
Acute pharyngitis	21/23 (91.3)	8/8 (100.0)	13/15 (86.7)	0.971
Acute tonsillitis	9/9 (100.0)	5/5 (100.0)	4/4 (100.0)	0.353
Acute bronchitis	92/100 (92.0)	29/33 (87.9)	63/67 (94.0)	0.455
Pneumonia	71/74 (95.9)	21/21 (100.0)	50/53 (94.3)	0.326
COPD exacerbation	34/40 (85.0)	18/19 (94.7)	16/21 (76.2)	0.040
Bronchoaspirative RTI	40/54 (74.1)	19/25 (76.0)	21/29 (72.4)	0.623
Influenza	11/23 (47.8)	2/5 (40.0)	9/18 (50.0)	0.375
COVID-19 infection	8/13 (61.5)	4/5 (80.0)	4/8 (50.0)	0.616
Other RTIs^a^	85/89 (95.5)	29/30 (96.7)	56/59 (94.9)	0.883
Total	413/533 (77.5)	146/187 (78.1)	267/346 (77.2)	0.811

COPD, chronic obstructive pulmonary disease; NA, not available; RTI, respiratory tract infection.

^a^ Any other RTI, that, according to professional judgment, cannot be classified under any of the above diagnoses.

Out of the total 413 cases of RTIs treated with antibiotics, 328 involved clear diagnoses, while the remaining cases were treated in residents with other types of RTIs. In 207 cases with a clear diagnosis (63.1%), antibiotic treatment was not indicated according to clinical guidelines.

### Predictive factors associated with antibiotic prescribing in residents with RTIs

As shown in Table 4, the most predictive factors for antibiotic use were the presence of confusion (odds ratio [OR] 4.72; p < 0.01), perceived demand for antibiotics (OR 3.83; p < 0.001), fever (OR 3.27; p < 0.01), increasing age (OR 2.58; p < 0.005), and increased sputum volume (OR 1.94; p < 0.05). In contrast, the presence of rhinorrhea and muscle pain were protective factors (OR 0.23; p < 0.001 and OR 0.35; p < 0.05, respectively). The presence of purulent sputum and breathlessness was associated with a higher likelihood of antibiotic prescribing, although these associations were not statistically significant (ORs of 2.02 and 1.87, respectively). Table 5 presents the factors associated with theoretically inappropriate antibiotic prescribing for RTIs with clear diagnoses among residents. Initiation of the antibiotic course outside the nursing home was significantly associated with a reduced likelihood of inappropriate antibiotic use (OR 0.26; p < 0.001). Hosmer–Lemeshow goodness-of-fit and collinearity were assessed in both tables, showing adequate model fit and no evidence of collinearity (Hosmer–Lemeshow p > 0.3 and all variance inflation factors <5).

**TABLE 4:** Predictive factors for the administration of antibiotics for residents with respiratory tract infections^a^.

Criteria	OR [CI 95%]	*P* value
Sex		
Male	1	​
Female	0.83 [0.47–1.44]	0.514
Age, years		
≤80	1	​
>80	2.58 [1.42–4.75]	0.002
Prior symptom duration, days		
≤2	1	​
>2	1.49 [0.83–2.72]	0.188
Antibiotic taken during the previous 15 days		
No	1	​
Yes	0.54 [0.28–1.07]	0.074
Perception of antibiotic demand		
No	1	​
Yes	3.83 [2.15–7.12]	<0.001
Fever		
No	1	​
Yes	3.27 [1.48–7.89]	0.005
Shaking chills		
No	1	​
Yes	1.65 [0.72–4.08]	0.254
Confusion		
No	1	​
Yes	4.72 [1.75–16.55]	0.005
Joint and muscle pains		
No	1	​
Yes	0.35 [0.15–0.86]	0.020
Cough		
No	1	​
Yes	1.17 [0.64–2.14]	0.616
Rhinorrhea		
No	1	​
Yes	0.23 [0.12–0.41]	<0.001
Odynophagia		
No	1	​
Yes	1.71 [0.76–4.10]	0.211
Dyspnea		
No	1	​
Yes	1.87 [0.98–3.69]	0.065
Increase in sputum volume		
No	1	​
Yes	1.94 [1.07–3.57]	0.030
Purulent sputum		
No	1	​
Yes	2.02 [0.78–5.97]	0.169
Bronchospasm		
No	1	​
Yes	1.48 [0.54–4.83]	0.476

CI, confidence interval; OR, odds ratio.

^a^ Otorrhea, tonsillar exudate, and tender cervical glands have been excluded due to the low number of observations.

**TABLE 5:** Risk factors associated with inappropriate antibiotic prescribing for respiratory tract infection^a^.

Criteria	OR [CI 95%]	*P* value
Age, years		
≤80	1	​
>80	0.98 [0.50–1.87]	0.946
Sex		
Man	1	​
Woman	1.21 [0.72–2.03]	0.475
Prior symptom duration, days		
≤2	1	​
>2	1.26 [0.76–2.13]	0.372
Antibiotic taken during the previous 15 days		
No	1	​
Yes	1.09 [0.58–2.10]	0.796
Perception of antibiotic demand		
No	1	​
Yes	0.67 [0.41–1.09]	0.108
Where the antibiotic course was initiated		
Nursing home	1	​
Other healthcare facility	0.26 [0.13–0.48]	<0.001

CI, confidence interval; OR, odds ratio.

^a^ Based on 328 antibiotic prescriptions for respiratory tract infections with a clear diagnosis.

## Discussion

This study highlights that 77.5% of RTI cases were treated with antibiotics, including a high proportion of infections typically considered viral in origin. In addition, antibiotics were also frequently prescribed for conditions that often resolve spontaneously without antimicrobial treatment. Another key finding is that more than 60% of antibiotic prescriptions were unnecessary based on clinical guidelines. Furthermore, the presence of certain clinical criteria increases the likelihood of both antibiotic prescribing, such as confusion, fever, and perceived demand for antibiotics. These findings highlight substantial room for improvement in antibiotic stewardship and offer insights into potential strategies to reduce inappropriate antibiotic use in nursing home settings.

More than three-quarters of all RTI cases in our study were treated with antibiotics. While antibiotics are warranted for residents with potentially serious infections such as pneumonia (Putot et al., 2025), growing evidence suggests that most RTIs, even in older adults, are self-limiting and do not benefit from antibiotic treatment. In fact, in many cases, antibiotic use in this population may cause more harm than good (Soraci et al., 2023). Clinical guidelines clearly advise against the use of antibiotics for viral infections such as influenza, the common cold, COVID-19, or acute bronchitis (Smith et al., 2017; Dilworth et al., 2022). Despite this, in our study, over 90% of acute bronchitis episodes were treated with antibiotics. Similarly, antibiotics are generally not indicated for other common self-limiting conditions such as acute rhinosinusitis, acute otitis media, or pharyngotonsillitis—unless a streptococcal infection is confirmed via a rapid antigen test, which is uncommon in older adults. Previous investigations in nursing homes have found that antibiotic prescribing was considered appropriate in only 42%–64% of cases (Lim et al., 2012; Peron et al., 2013; Zimmer et al., 1986; Stuart et al., 2012). The results of this observational study show a high rate of unnecessary antibiotic use, though it remains within the range reported in earlier studies.

The fact that the most predictive factor for unnecessary antibiotic use was initiation within the nursing home is particularly concerning. It suggests that processes and decision-making that occur on site—rather than in hospitals or outpatient clinics—materially increase the risk of inappropriate treatment. Several mechanisms may explain this association (CDC, 2015). First, diagnostic uncertainty is common in nursing home residents (atypical presentations, limited access to imaging and lab tests), and this uncertainty frequently drives empiric prescribing. Evidence shows that routine testing and treatment of nonspecific symptoms are important sources of unnecessary UTI prescribing in care homes. Second, nursing staff critically influence the initiation pathway: nurses and nursing aides are frontline in assessing subtle changes, communicating with prescribers (often by phone), and recommending testing or treatment; their perceptions, anxiety about clinical deterioration, and patterns of communication with physicians strongly affect antibiotic starts. Third, compared with other settings there is wide variation in prescribing thresholds and fewer immediate diagnostic supports (e.g., onsite CRP, chest X-ray), so clinicians may adopt a lower threshold to start antibiotics “to be safe.” The results of our study clearly show a very limited use of rapid tests, with COVID-19 tests being the most frequently used, but in only one-quarter of cases. In most instances, other rapid tests, such as those for influenza or CRP, are not available in this setting. Finally, organizational factors, such as staffing levels, physician visit patterns, local culture, and incentives, create heterogeneity between facilities and can perpetuate routine unnecessary prescribing (Fleming et al., 2014). This finding emphasizes the need for antimicrobial stewardship interventions to be specifically tailored to the practices and needs of nursing home staff.

One major driver of antibiotic overprescription is the difficulty of distinguishing viral from bacterial infections based on clinical criteria alone, and this is aggravated by the lack of rapid diagnostic tests in this setting, as observed in this study. Only two conditions—COPD exacerbations and acute tonsillitis—have well-established clinical predictors that reliably indicate when antibiotics are warranted (Anthonisen et al., 1987; Centor et al., 1981). Prior research shows that clinicians often rely on specific findings in suspected acute tonsillitis, such as tonsillar exudate, to guide treatment decisions (Llor et al., 2010). However, in our study the number of pharyngitis or tonsillitis cases was very low, limiting our ability to assess how such clinical features influenced prescribing in this setting. Several studies have shown that sputum-related findings—particularly increased volume and purulence—predict antibiotic prescribing (Chen et al., 2020; Llor et al., 2013), a pattern also observed in our study. Although purulent sputum may suggest bacterial infection in COPD exacerbations, it is an unreliable indicator in acute bronchitis, which was markedly more common in our population. Evidence has long demonstrated that antibiotics offer no benefit over placebo for acute bronchitis, even when sputum is purulent (Smith et al., 2017; Stott and West, 1976). For COPD exacerbations, a bacterial origin is only likely when at least two Anthonisen criteria are present (increased dyspnea, sputum volume, and sputum purulence), and only then are antibiotics recommended. Despite this, empirical prescribing remains widespread in Spain; one survey reported antibiotics in 90% of COPD exacerbations (Miravitlles et al., 1999), slightly higher than in our study, possibly because chronic bronchitis exacerbations were included in the same category. Notably, dyspnea was not associated with antibiotic prescribing in our data. Fever also strongly influenced prescribing. While it supports bacterial etiology in pharyngotonsillitis, its interpretation in lower RTIs is often mistaken. Although commonly linked to pneumonia, fever occurs across many RTIs and is not a criterion for antibiotic treatment in COPD exacerbations. Once pneumonia is excluded, high fever may even suggest viral infection (Lieberman et al., 2003). Confusion was another factor associated with antibiotic use in our study, consistent with previous findings, particularly among residents with dementia (D'Agata and Mitchell, 2008). These patients are also more likely to receive broad-spectrum agents such as quinolones compared with residents without cognitive impairment. Previous studies in our country have also demonstrated this association (Carter et al., 2017; Llor et al., 2014). Clinicians are more likely to prescribe antibiotics when they perceive that patients or relatives expect them, even without an explicit request. Often, simply sensing that antibiotics are desired can influence prescribing decisions (Domen et al., 2025). Patients frequently hold misconceptions about the benefits of antibiotics for RTIs, commonly believing they lead to faster recovery (Almeshal et al., 2024).

This study has several limitations that warrant consideration. Although nursing home staff primarily comprises nurses, the authority to prescribe antibiotics resides with physicians. In the Spanish context, however, physicians in nursing homes are typically external consultants who are not present during nights or weekends, whereas nursing staff are continuously available. This organizational structure may influence prescribing practices. Furthermore, the data collection template did not include information on residents’ comorbidities, despite the fact that underlying chronic conditions can significantly influence clinical decision-making and antibiotic prescribing in older adults. This limitation may have prevented us from fully capturing important factors that contribute to treatment choices in this population. Nonetheless, the large sample size in this study likely mitigates the risk of significant bias arising from this omission. Similarly, while not all signs and symptoms of RTIs were captured, owing to the pragmatic constraints of conducting such a study in routine clinical settings, the registry still included 88 variables. Another notable limitation is the absence of clinical outcome measures. However, the registration forms did include data on hospital referrals, offering a partial proxy for clinical impact. Additionally, the process of self-registration may have influenced prescribing behavior. While treatment decisions should ideally follow diagnostic assessment, in routine clinical practice these processes are frequently intertwined. As previously noted in other studies (Howie, 1972; Xingrong et al., 2022), decisions regarding antibiotic therapy may be made concurrently with, or even prior to, the establishment of a formal diagnosis. It cannot be guaranteed that all nursing home staff collected data consistently over the full three-month period; however, this limitation is not expected to have affected the overall results of the study. Despite these limitations, the data collection process was designed to be minimally disruptive. The registration forms required staff only to check pre-defined criteria, without the need for narrative input, thereby allowing participating nurses to maintain their usual workflows during the study period.

This cross-sectional study provides valuable insights into antibiotic use for RTIs within the real-world context of nursing homes. The findings highlight the persistent issue of antibiotic overprescribing, with only slightly more than one-third of prescriptions deemed appropriate. The fact that treatments initiated in nursing home settings are more frequently unnecessary makes the implementation of antimicrobial stewardship interventions in this setting essential. The implementation of antimicrobial stewardship programs in this setting has been shown to effectively reduce inappropriate antibiotic use (Tandan et al., 2022); however, a recent narrative review revealed that the impact of such programs varies considerably across long-term care facilities (Falcone et al., 2019). In most cases, the decision to prescribe antibiotics is based solely on clinical criteria, as rapid diagnostic tests are either not routinely employed or are not readily accessible. Evidence suggests that improving access to these tests in nursing homes could help reduce unnecessary antibiotic prescribing (Boere et al., 2021). Furthermore, studies from other settings have demonstrated that rapid tests can be helpful in managing patient expectations, especially when patients or their relatives request antibiotics. In this context, our results may also support efforts to educate and reassure residents and their families when antibiotics are not clinically indicated.

In conclusion, the findings of this study open new avenues for the development and implementation of targeted strategies to promote more rational antibiotic use in nursing homes. These may include incorporating simple decision-support tools tailored to the needs of nursing home staff, expanding access to rapid diagnostic tests to support more accurate clinical decision-making, and providing targeted training for personnel involved in assessing RTIs. Together, such measures could help translate our findings into practical improvements in antimicrobial stewardship within this care setting.

## Data Availability

The raw data supporting the conclusions of this article will be made available by the authors, without undue reservation.
